# Nucleation dynamics of single crystal WS_2_ from droplet precursors uncovered by *in-situ* monitoring

**DOI:** 10.1038/s41598-019-49113-0

**Published:** 2019-09-10

**Authors:** Chao Li, Tomoya Kameyama, Tomoyuki Takahashi, Toshiro Kaneko, Toshiaki Kato

**Affiliations:** 1 Department of Electronic Engineering, Tohoku University, 980-8579 Sendai, Japan; 2 JST-PRESTO, Tohoku University, 980-8579 Sendai, Japan

**Keywords:** Two-dimensional materials, Two-dimensional materials

## Abstract

Transition metal dichalcogenides (TMDs) attract intence attention due to its unique optoelectrical features. Recent progress in production stage of TMD enables us to synthesis uniform and large area TMD with mono layer thickness. Elucidation of growth mechanism is a challenge to improve the crystallinity of TMD, which is regargeded as a next crutial subject in the production stage. Here we report novel diffusion and nucleation dynamics during tungsten disulphide (WS_2_) growth. The diffusion length (*L*_d_) of the precursors have been measured with unique nucleation control methods. It was revealed that the *L*_d_ reaches up to ~750 μm. This ultra-long diffusion can be attributed to precursor droplets observed during *in-situ* monitoring of WS_2_ growth. The integrated synthesis of >35,000 single crystals and monolayer WS_2_ was achieved at the wafer scale based on this model. Our findings are highly significant for both the fundamental study of droplet-mediated crystal growth and the industrial application of integrated single-crystal TMDs.

## Introduction

Transition metal dichalcogenides (TMDs) are among the most well-known layered materials. They have various features that are desirable in semiconductors, including stable neutral and charged excitons, valley polarisation capability, and superconductivity^1–3^. Recent progress with molten salt-assisted growth^4,5^ in the production stage enabled us to synthesise uniform polycrystalline films on a large scale. Attaining better crystallinity in single crystal films with large domain sizes, uniform edge structures, and fewer vacancies is our next challenge. For this purpose, a deep understanding of the TMD crystal growth mechanism is crucial. The effects of the sulfur/molybdenum (S/Mo) ratio^6^ in MoS_2_, the step edge of the substrate^7^, the carrier gas^8^ and nucleation promoters^9^ have been investigated. A theoretical model has also been established using a thermodynamic approach^10,11^. However, little is known about TMD growth dynamics, their precursors and nucleation in particular, which are important for the synthesis of high-quality single crystals. Herein we report novel diffusion and nucleation dynamics during tungsten disulfide (WS_2_) growth. Accurate nucleation-controlled growth enabled us to determine the diffusion length (*L*_d_) of the precursors. *L*_d_ reached ~750 μm, nearly two orders of magnitude longer than that of conventional semiconductors such as Si^12^, GaAs^13^ and SiC^14^. This ultra-long diffusion was attributed to precursor droplets observed during *in-situ* monitoring of WS_2_ growth. The integrated synthesis of >35,000 single crystals and monolayer WS_2_ was achieved at the wafer scale based on this model. Our findings are highly significant for both the fundamental study of droplet-mediated crystal growth and the industrial application of integrated single-crystal TMDs.

**Figure 1:**
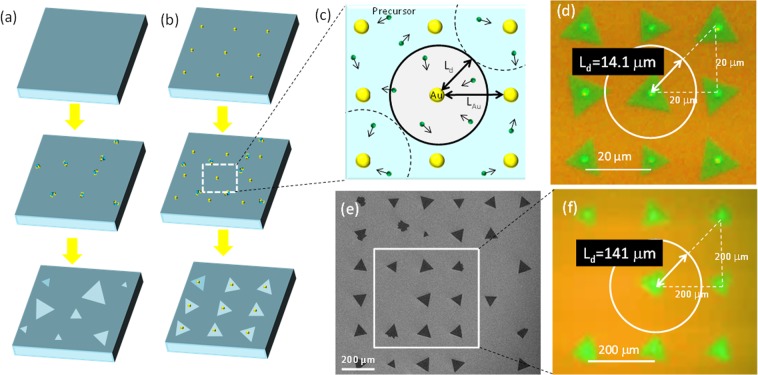
Nucleation control with Au dots. Schematic illustration of TMD growth (**a**) without and (**b**) with Au nucleation centre. (**c**) Correlation between *L*_d_ and *L*_Au_. (**d**,**f**), Optical microscope and (**e**) SEM images of monolayer and single WS_2_ crystals grown on Au pre-patterned substrate, where *L*_Au_ equals (**d**) 20 μm and (**e**,**f**) 200 μm.

Control of nucleation sites^15,16^ is useful for studying the growth dynamics of 2D crystals. In this study, we used Au dots to precisely control WS_2_ nucleation and site density (Fig. 1a–c, Supplementary Fig. 1). Figure 1d shows the results of WS_2_ synthesis on a substrate containing an array of Au dots, for which the distance between the centres of the Au dots (*L*_Au_) was 20 μm. Triangular WS_2_ crystals were grown at the Au sites, thereby demonstrating that precise control of the nucleation sites and their density was possible.

Diffusion length (*L*_d_), an important kinetic parameter of crystal growth, was determined experimentally (Fig. 1c). WS_2_ growth was observed only at Au sites, while the SiO_2_ substrate remained bare between the Au dots (Fig. 1d). This suggested two nucleation mechanisms were possible. If nearly all of the precursor material delivered to the substrate from the vapor phase was effectively trapped by the Au dots and used for WS_2_ growth, *L*_d_ could be estimated by Eq. (1).

1 $${{\rm{L}}}_{{\rm{d}}}\approx \sqrt{2}{L}_{Au}/2.$$

Interestingly, nucleation of single WS_2_ crystals was observed only at Au sites even when *L*_Au_ ≤ 200 μm (Fig. 1e,f), indicating the maximum *L*_d_ would be ≥140 μm. This was surprising, considering the *L*_d_ of conventional semiconductor materials such as Si^12^, GaAs^13^ and SiC^14^ ranges from 0.2 to 30 μm (Supplementary Table 1). It should be noted that if precursors could immediately desorb from the substrate before being trapped by Au, *L*_d_ determined by eq. (1) could be an overestimate.

**Figure 2:**
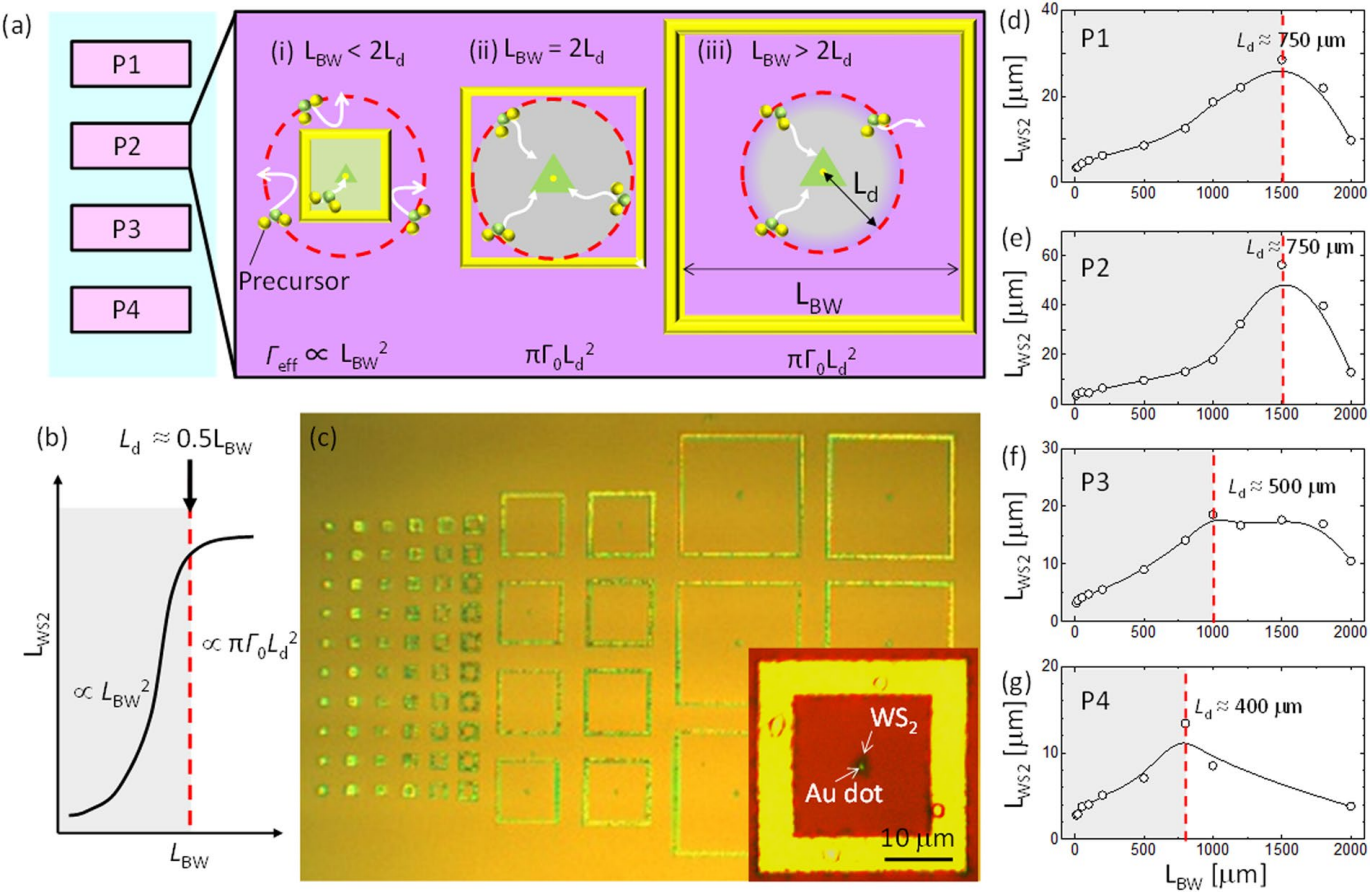
Direct measurement of *L*_d_. (**a**) The growth region on a single Au dot is surrounded by a square diffusion barrier made by Au. The relationships between *L*_WS2_ and *L*_BW_ are (i) *L*_BW_ < 2*L*_d_; (ii) *L*_BW_ = 2*L*_d_; and (iii) *L*_BW_ ≫ 2*L*_d_. The effective influx contributing to WS_2_ growth (Γ_eff_) is governed by (i) *L*_BW_^2^ and (ii, iii) *L*_d_^2^. (**b**) The predicted relationship between *L*_WS2_ and *L*_BW_. (**c**) Optical microscope image showing substrate surface after WS_2_ growth with the diffusion barriers. Inset in (**c**) shows the high magnification image of typical WS_2_ grown inside of diffusion barrier. Plots of experimental *L*_WS2_ vs. *L*_BW_ in different regions of the substrate: (**d**) P1, (**e**) P2, (**f**) P3 and (**g**) P4, where the temperature was 721, 698, 675, 654 °C, respectively.

To more accurately determine *L*_d_ and observe ultra-long precursor diffusion, we placed diffusion barriers around the Au dots (Fig. 2a). Crystal growth was terminated at the initial growth stage, when the length of each side of the triangular WS_2_ crystal (*L*_WS2_) increased with growth time. *L*_WS2_ was related to (*Γ*_eff_*t*)^0.5^ in our model, where *Γ*_eff_ and *t* were the effective precursor flux to the growth edge of WS_2_ and growth time, respectively. *Γ*_eff_ was proportional to π*Γ*_0_*L*_d_^2^, where *Γ*_0_ was the influx of vapor-phase precursors to the substrate per unit area (Supplementary Fig. 2). *Γ*_eff_ was influenced by the length of the diffusion barrier wall (*L*_BW_), so *L*_WS2_ would change as a function of *L*_BW_ (Fig. 2a,b). Based on this model, we predicted a region in which *L*_WS2_ growth would transition from a quadratic function of *L*_BW_ to a saturated state (Fig. 2b). *L*_BW_ would equal 2*L*_d_ at the inflection point in Fig. 2b.

For the experiment, square diffusion barriers of various sizes were placed on the substrate. Each diffusion barrier was centred around a single Au dot (Fig. 2c). As expected, there was an obvious relationship between *L*_WS2_ and *L*_BW_ (Fig. 2d–g). When *L*_BW_ was small, *L*_WS2_ increased with *L*_BW_ and reached a point of saturation after reaching the critical threshold of *L*_BW_. This indicated the size of WS_2_ crystals was governed by diffusion, and that *L*_d_ could be determined by the *L*_BW_ threshold. We found that variation in *L*_d_ depended on the position of nucleation on the substrate (P1, P2, P3, P4) within the region where the temperature decreased from ~721 °C to ~654 °C. The maximum *L*_d_ of ~750 μm was observed at 721 °C (P1) and 698 °C (P2) (Fig. 2d,e). The observed ultra-long diffusion was consistent with estimations based on *L*_Au_ (Fig. 1f). This was the first experimental determination of *L*_d_ during TMD growth. The Arrhenius-type correlation can be observed btween P2 and P4 positions, where activation energy can be estimated as ~1.1 eV (Supplementaly Fig. S3). This shows the thermal activation of precursors can enhance the diffusion by overcoming the diffusion barriers. Since the *L*_d_ is almost same between P1 and P2, which may be decided by the balance between enhancement of *L*_d_ with thermal activation and decreasing *L*_d_ due to acceralation of desorption from the substrate.

Hopping transport is the predominating model for conventional semiconductors, in which precursors jump between nearest-neighbour (NN) stable sites. Diffusion occurs over a few micrometres within the limited traveling time^12–14^. When we tried to explain the ultra-long diffusion of WS_2_ precursors with this model, the distance covered in a single jump was over 100 times larger than the distance between NN sites. Although ‘long jumping’ during the diffusion of W on a substrate surface has been reported, the longest jumps were only a few times longer than the distance between NN sites^17,18^. Ultra-long jumps (100 × NN length) have not been reported for any materials, suggesting that WS_2_ precursors diffuse by a different mechanism. A possible mechanism for ultra-long precursor diffusion will be discussed later.

**Figure 3:**
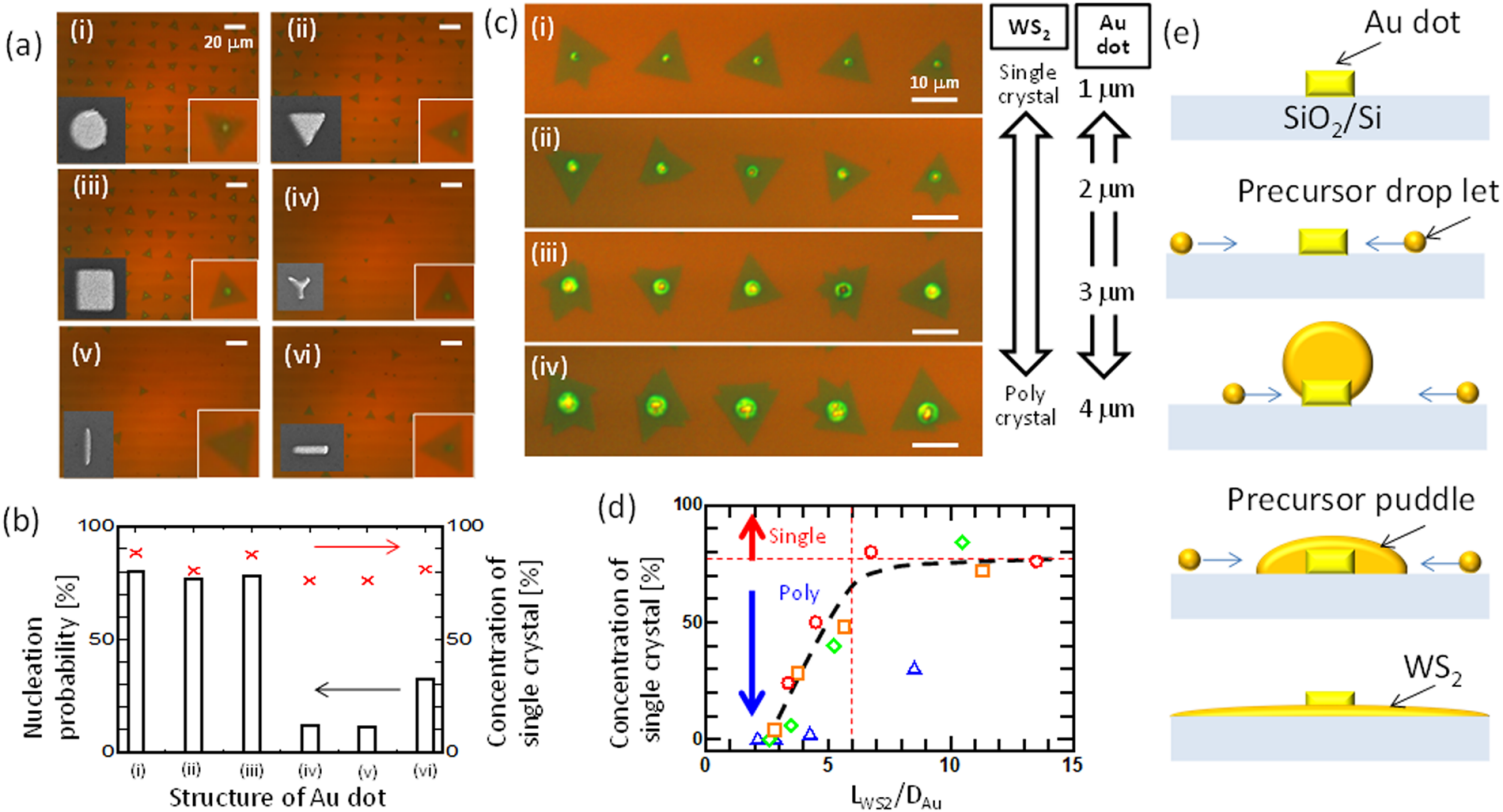
Nucleation of WS_2_ at Au sites. (**a**) Optical microscope images of WS_2_ grown from various Au dot structures (i–vi). Left and right of inset in (i–vi) show the SEM image of Au dot structures used for each growth and typical WS_2_ grown from each Au dot, respectively. (**b**) Dependence of WS_2_ growth shape on Au dot size. (**c**) Optical microscope images of WS_2_ grown on Au dots of varying size (*D*_Au_): (i) 1 μm, (ii) 2 μm, (iii) 3 μm and (iv) 4 μm. (**d**) Concentration of single WS_2_ crystals as a function of *L*_WS2_/*D*_Au_. Growth temperatures: ○ 816 °C, □ 800 °C, ◊ 795 °C, Δ 773 °C. (**e**) Schematic illustration of droplet-induced nucleation dynamics.

To reveal WS_2_ growth dynamics, understanding the nucleation phase is important. We varied Au dot shape and diameter (*D*_Au_) in a combinatorial experiment (see Methods) to gain insight into the nucleation dynamics of WS_2_ (Fig. 3a). There was a strong correlation between nucleus structure and nucleation probability (Fig. 3a,b and Supplementary Fig. S4). Surprisingly, however, the crystallinity of monolayer WS_2_ was independent of Au dot shape, and WS_2_ single crystals grew on circular, triangular, square, and linear bar-shaped sites. We then varied the *D*_Au_ of circular Au dots, and WS_2_ growth was carried out at different temperatures using a combinatorial method (see Methods, Supplementary Fig. S5). The single WS_2_ crystal concentration was higher when growth occurred on smaller Au dots (*D*_Au _~ 1 μm) than it was when *D*_Au_ ≈ 4 μm (Fig. 3c). We then changed *L*_WS2_ by adjusting the growth temperature (Supplementary Figs S5 and S6). When we plotted the single crystal concentration as a function of *L*_WS2_/*D*_Au_, a clear correlation was observed. The concentration of WS_2_ single crystals increased with *L*_WS2_/*D*_Au_ and reached saturation when (L_WS2_/*D*_Au_) > 6 (Fig. 3d).

Based on these results, we concluded (i) precursor diffusion length was ultra-long (~750 μm); (ii) single crystals could grow on Au dot structures with various shapes; and (iii) there was a clear correlation between single crystal concentration and L_WS2_/*D*_Au_. We could then propose a plausible model for the diffusion and nucleation of precursors (Fig. 3e). Since the shapes of the Au dots were not sensitive to the WS_2_ structure (Fig. 3a,b), we surmised that a circular precursor puddle could form around the Au dot prior to the initiation of 2D growth. Polycrystalline WS_2_ would grow when the diameter of the precursor puddle was less than *D*_Au_ (Supplementary Fig. S6). Following this logic, precursors would diffuse on the substrate in droplet form rather than as single molecules. This could explain the ultra-long diffusion of precursors on the SiO_2_ substrate (Fig. 2). It is known that interaction between droplets and the substrate occurs via physisorption rather than chemisorption^19,20^. This is completely different from atomic and molecular diffusion, where NN hopping is dominant due to strong chemical interactions with the substrate surface^12–14^. Physisorbed droplets move easily on the SiO_2_ surface for a relatively long time^19,20^, which would enable ultra-long diffusion. The stability of droplets can be evaluated by the Young-Laplace (YL) equation^21^:

2 $$\Delta P=2\gamma /r.$$

Here, Δ*P* is the difference between the droplet pressure (*P*_in_) and vapor pressure (*P*_out_) at the interface; *γ* is the surface tension of the droplet; and *r* is the droplet radius. For the droplet to be stable, Δ*P* should be as small as possible. This means that materials with low *γ* are more stable, especially at the nanoscale (*r* < 500 nm). The *γ* of molten metal droplets can be reduced by increasing the temperature^22^, reducing droplet size^23^, and mixing with oxygen^24^. It can be surmised that nanoscale droplets containing W_x_S_y_O_z_ at high temperatures would exhibit very low surface tension, making them candidate precursor droplets. Since the physical properties of nanoscale droplets themselves are not yet fully understood^25^, the identification of precursor droplet components is an important step for achieving a detailed understanding of WS_2_ growth dynamics.

**Figure 4:**
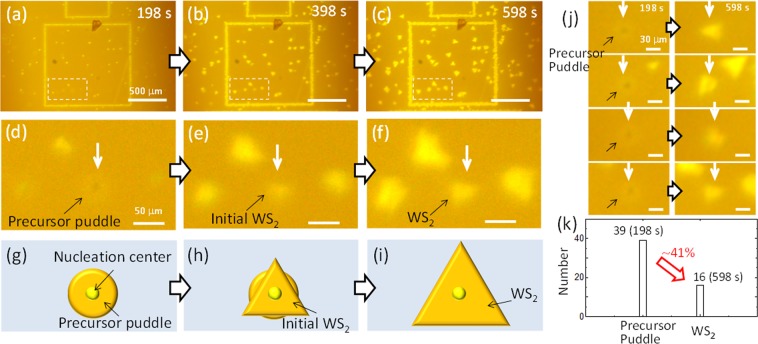
*In-situ* monitoring of WS_2_ growth. Low- and high-magnification optical microscope images from *in-situ* monitoring of WS_2_ growth at (**a**,**d**) 198 s, (**b**,**e**) 398 s and (**c**,**f**) 598 s. Where growth time: 0 s corresponds to the time when all process temperatures (main furnace, sulphur oven, and AH) were stabilized. Schematic illustration of nucleation model from (**g**) droplet puddle to (**h**,**i**) 2D WS_2_. (**j**), Optical microscope images of typical precursor puddles transferred to WS_2_. (**k**), Number of precursor puddles at 198 s and WS_2_ formed from the precursor puddles at 598 s.

To confirm the unique growth dynamics of droplet-induced nucleation, we attempted to observe the nucleation phase directly via *in-situ* monitoring (see Methods, Supplementary Fig. S7). The increase of *L*_WS2_ was observed in real time, confirming that *in-situ* monitoring of WS_2_ crystal growth was possible (Fig. 4a–c, Supplementary Fig. S8, Supplementary Movie). To the best of our knowledge, this is the first result realizing the *in-situ* monitoring of TMD growth as real time optical images. When we carefully examined the nucleation with high-magnification images, an interesting transformation was observed (Fig. 4d–f). At the initiation of growth, circular structures formed around the naturally existing nucleation centre (Fig. 4d,g). These changed to triangular shapes during the growth stage (Fig. 4e,f,h,i). This was consistent with the model shown in Fig. 3e, indicating the precursor puddle formed just after nucleation around the nucleation centre. Similar transformations from precursor puddles to WS_2_ were observed at many nucleation sites (Fig. 4j, Supplementary Fig. S9). This established the veracity of droplet-induced growth dynamics (Figs 3e and 4g–i). We also found that ~41% of the precursor puddles transformed to WS_2_ during the growth stage. The state of the precursor puddle and the driving force causing the transformation from liquid to solid should be important factors in deciding this transformation. Further studies are needed to clarify these fundamental subjects. It should be noted that some WS_2_ crystals began to grow without the obvious formation of a precursor puddle, suggesting several growth models could apply to the nucleation of TMD. Recently, a vapor-liquid-solid (VLS) growth model was reported for the catalyst-guided growth of MoS_2_ nanoribbons^26^. In the growth of 2D WS_2_ demonstrated in our study, it was not a catalyst but the precursor itself in liquid form that diffused over long distances. This differed significantly from the mechanism of nanoribbon growth.

**Figure 5:**
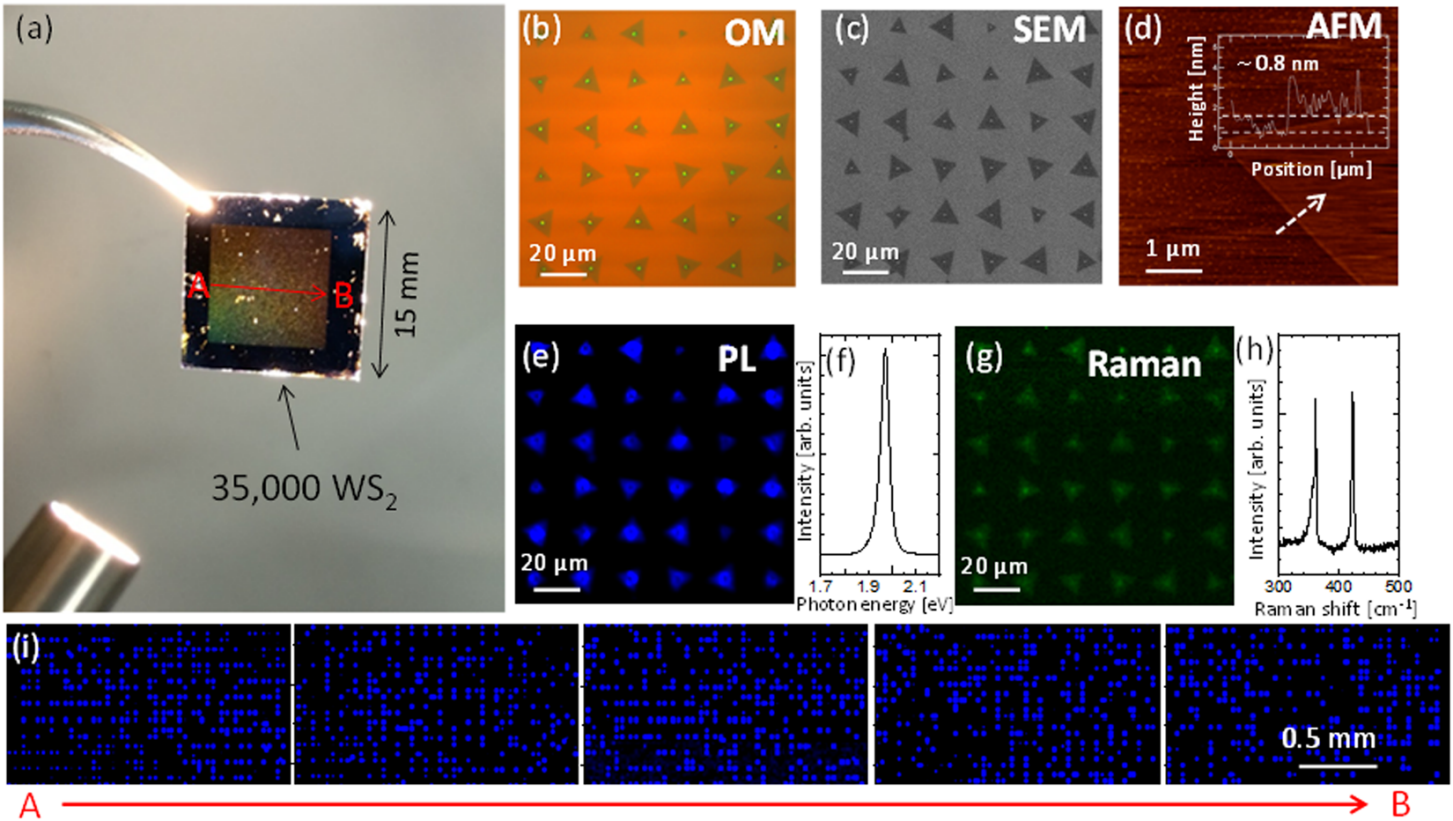
Position selective large-scale synthesis of single WS_2_ crystals. (**a**) Optical microscope image of WS_2_ array grown on a 1.5 cm × 1.5 cm wafer substrate. Images of WS_2_ array grown on Au dots: (**b**) optical microscope, (**c**) SEM, (**d**) AFM, (**e**) PL intensity map, (**g**) Raman intensity map. Raw (**f**) PL and (**h**) Raman scattering spectra of WS_2_ grown on Au dot. (**i**) Large-scale WS_2_ PL intensity map from A to B on the wafer in (**a**).

Based on our growth model, precise adjustments of *D*_Au_, *L*_Au_ and growth temperature were made in a combinatorial experiment (see Methods, Supplementary Fig. S10). These systematic adjustments produced the conditions required for integrated synthesis of single WS_2_ crystals, which was governed by a balance between nucleation probability and single crystal growth (Supplementary Fig. S11). Under the most suitable growth conditions, integrated WS_2_ was grown on a 1.5 cm × 1.5 cm region that covered the entire substrate (Fig. 5a). Optical microscope (Fig. 5b) and scanning electron microscope (SEM) (Fig. 5c) images indicated that triangular WS_2_ crystals grew on the Au dots in high yields (>87%). Measurement by atomic force microscopy (AFM) revealed the thickness of WS_2_ was approximately 0.8 nm (Fig. 5d). The crystalline WS_2_ shown in the photoluminescence (PL) intensity map in Fig. 5e exhibited bright PL with a sharp emission peak at 1.97 eV (Fig. 5f). The Raman spectra of WS_2_ contained two peaks separated by 61 cm^−1^ (Fig. 5g,h), indicating WS_2_ grown by this method was a monolayered, single-crystal structure^1^. PL intensity mapping was performed over a large area between A and B in Fig. 5a, and uniform PL was observed across the whole 1.5 cm width of the substrate (Fig. 5i). These results indicated that >35,000 single WS_2_ crystals could be grown with accurate position control. It should be mentioned that the Au dot is remained in the ceter of WS_2_ evern after the growth. Detailed analysis around Au dot is shown in supporting information (Fig. S3). Although the PL can be quenched around the Au dot region, the Au dot size can be minimized below 300 nm through the adjustment of growth temperature (Fig. S10). In the case of relatively long channel device such as thin film transistors, the Au dot below 300 nm may be considered as a small single defect, which may not cause significant depression of whole device performance.

In summary, we have revealed the detailed growth dynamics of monolayer and single-crystal WS_2_. Through control of nucleation with Au dots and diffusion barriers, the *L*_d_ reach up to ~750 µm, which was almost two orders of magnitude longer than that of conventional semiconductor materials. The combinatorial experiment demonstrated that ultra-long diffusion could be explained by precursor droplet formation. Precursor droplets collected around the Au dots to form precursor puddles before 2D growth commenced. The balance between the size of the puddle and the Au dot was important in determining the probability of single WS_2_ crystal growth. *In-situ* monitoring established the accuracy of droplet-induced growth dynamics. Based on this model, integrated synthesis of monolayer and single-crystal WS_2_ was realised at the wafer scale. Over 35,000 single crystals and monolayer WS_2_ were grown on a 1.5 cm^2^ substrate. This insight into the growth dynamics of single-crystal WS_2_ may serve as an impetus to move the study of TMDs from fundamental research to practical applications. Controlling the diffusion of precursor droplets may provide an alternate means of controlling TMD crystallinity.

## Methods

### Preparation of Au dot arrays

The Au dot arrays were fabricated on a SiO_2_/Si substrate by electron beam lithography (EBL) on an ELS-7500 EBL system (ELIONIX Inc., Japan) and thermal evaporation of Au (see Supplementary Information for more details). The thickness of each Au dot was fixed at 50 nm.

### Chemical vapor deposition (CVD)

WS_2_ was synthesised by thermal CVD using WO_3_ as a tungsten source. Ar was used as the carrier gas at a flow rate of 150–450 sccm. Sulphur (0.5 g) was placed in the CVD oven (Supplementary Fig. S7), and WO_3_ (40 mg) on a quarts boat was set 15 cm downstream in the centre of the CVD furnace. For *in-situ* monitoring, NaCl (6 mg) was mixed into the WO_3_ to enhance evaporation.

### Combinatorial experiment

An array of Au dots 100, 300, 500, 750 and 1000 nm in diameter was prepared on a large SiO_2_/Si substrate (Supplementary Fig. S10) placed 7 cm from the centre of the furnace. Since the temperature in this section decreased sharply from 860 to 700 °C within a 2-cm region with 975 °C of centre of furnace temperature, it was possible to control the growth temperature and Au dot size/shape in the same experiment.

### Raman an PL mapping

A J/Y Raman/PL system was used for Raman and PL mapping. He/Ne (632.8 nm) and Ar (488 nm) lasers were used for excitation. Mapping was performed in steps of 200–500 nm.

### *In-situ* monitoring

The optical microscope was set above the quartz tube between the main electrical furnace and additional heater (AH). To maintain the elevated temperature of the substrate, an AH was placed outside the main electrical furnace. The substrate was set between the main furnace and the heater, enabling optical observation of the substrate surface during CVD growth (Supplementary Fig. S7).

## Supplementary information

Supplementary Dataset

Supplementary Information

## Data Availability

The authors declare that the data supporting the findings of this study are available within the article and its supplementary figures, table, and movie files.
