# Author Correction: Nucleation dynamics of single crystal WS_2_ from droplet precursors uncovered by *in-situ* monitoring

**DOI:** 10.1038/s41598-020-58026-2

**Published:** 2020-01-31

**Authors:** Chao Li, Tomoya Kameyama, Tomoyuki Takahashi, Toshiro Kaneko, Toshiaki Kato

**Affiliations:** 1 Department of Electronic Engineering, Tohoku University, 980-8579 Sendai, Japan; 2 JST-PRESTO, Tohoku University, 980-8579 Sendai, Japan

Correction to: *Scientific Reports* 10.1038/s41598-019-49113-0, published online 10 September 2019

A Supplementary Movie was omitted from the original version of this Article. This has been corrected in the HTML version of the Article; the PDF version was correct at the time of publication.

