# Supplementary material for: Nucleation dynamics of single crystal WS2 from droplet precursors uncovered by in-situ monitoring
Source: Sci Rep. 2019 Sep 10;9:12958. doi: 10.1038/s41598-019-49113-0 (PMC6736981; doi:10.1038/s41598-019-49113-0)
Supplement: Supplementary file 1 — Supplementary Dataset [file 41598_2019_49113_MOESM1_ESM.pdf]

Supplemental Information for

Nucleation dynamics of single crystal WS_2 from droplet precursors uncovered by in-situ monitoring

Chao Li[1], Tomoya Kameyama[1], Tomoyuki Takahashi[1], Toshiro Kaneko[1] & Toshiaki Kato*[1,2]

[1]Department of Electronic Engineering, Tohoku University, 980-8579 Sendai, Japan

[2]JST-PRESTO, Tohoku University, 980-8579 Sendai, Japan

Supplementary Figure 1 │ Au dot structures.

Supplementary Figure 2 │ Schematic illustration of precursor influx.

Supplementary Figure 3 │Temperature dependence of L_d.Supplementary Figure 4 │ Effects of Au dot shape.

Supplementary Figure 5 │ Effects of Au dot size.

Supplementary Figure 6 │ Effect of D_{Au} on WS_2 single crystal growth.

Supplementary Figure 7 │ In-situ monitoring system for WS_2 growth.

Supplementary Figure 8 │ Detailed results from in-situ monitoring.

Supplementary Figure 9 │ Transformation from precursor puddle to WS_2.

Supplementary Figure 10 │ Combinatorial experiment for position-selective synthesis of WS_2.

Supplementary Figure 11 │ WS_2 single crystal growth window on Au dots.

Supplementary Table 1| L_d of conventional semiconductor materials.

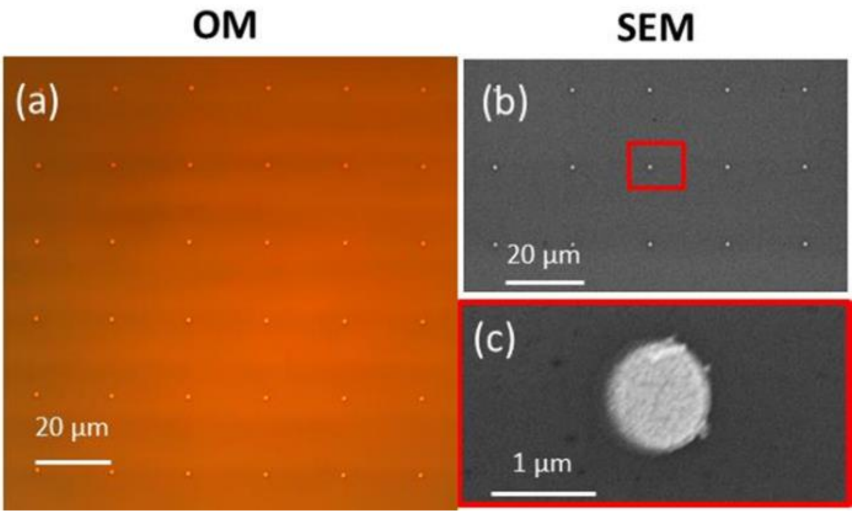

Supplementary Figure 1 | Au dot structures. Typical (**a**) optical microscope, (**b**) low-magnification SEM and (**c**) high-magnification SEM images of Au dots fabricated by electron beam lithography.

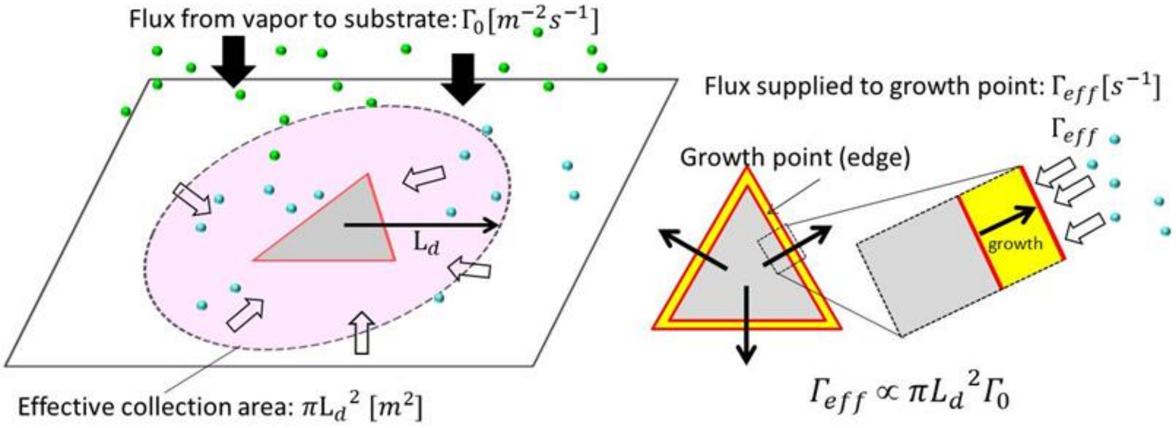

Supplementary Figure 2 │ Schematic illustration of precursor influx. Precursors transfer from the vapor phase to the substrate with flux Γ_0. Precursor droplets diffuse on the substrate within the range L_d, such that the precursors supplied within the area $\pi L_d{}^2$ around the nucleation centre (pink region at left) can be effectively used for the WS_2 growth. Since the TMD growth point can be considered the edge of the crystal, growth speed should be proportional to the precursor flux supplied to each growth point (Γ_{eff}), which is proportional to $\pi L_d{}^2 \Gamma_0$.

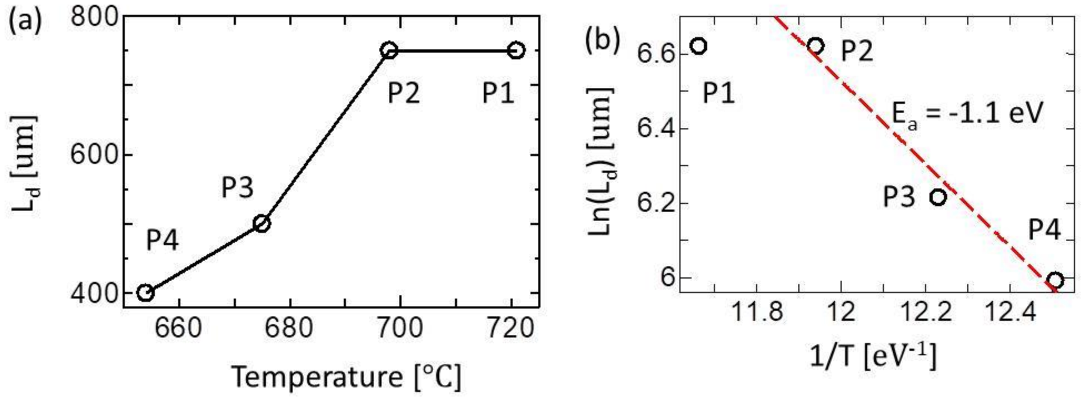

Supplementary Figure 3 │ Temperature dependence of $L_\mathbf{d}$. (a) L_d vs Temperature obtained from different positions (P1-P4). (b) Arrhenius-type plot of data shown in (a).

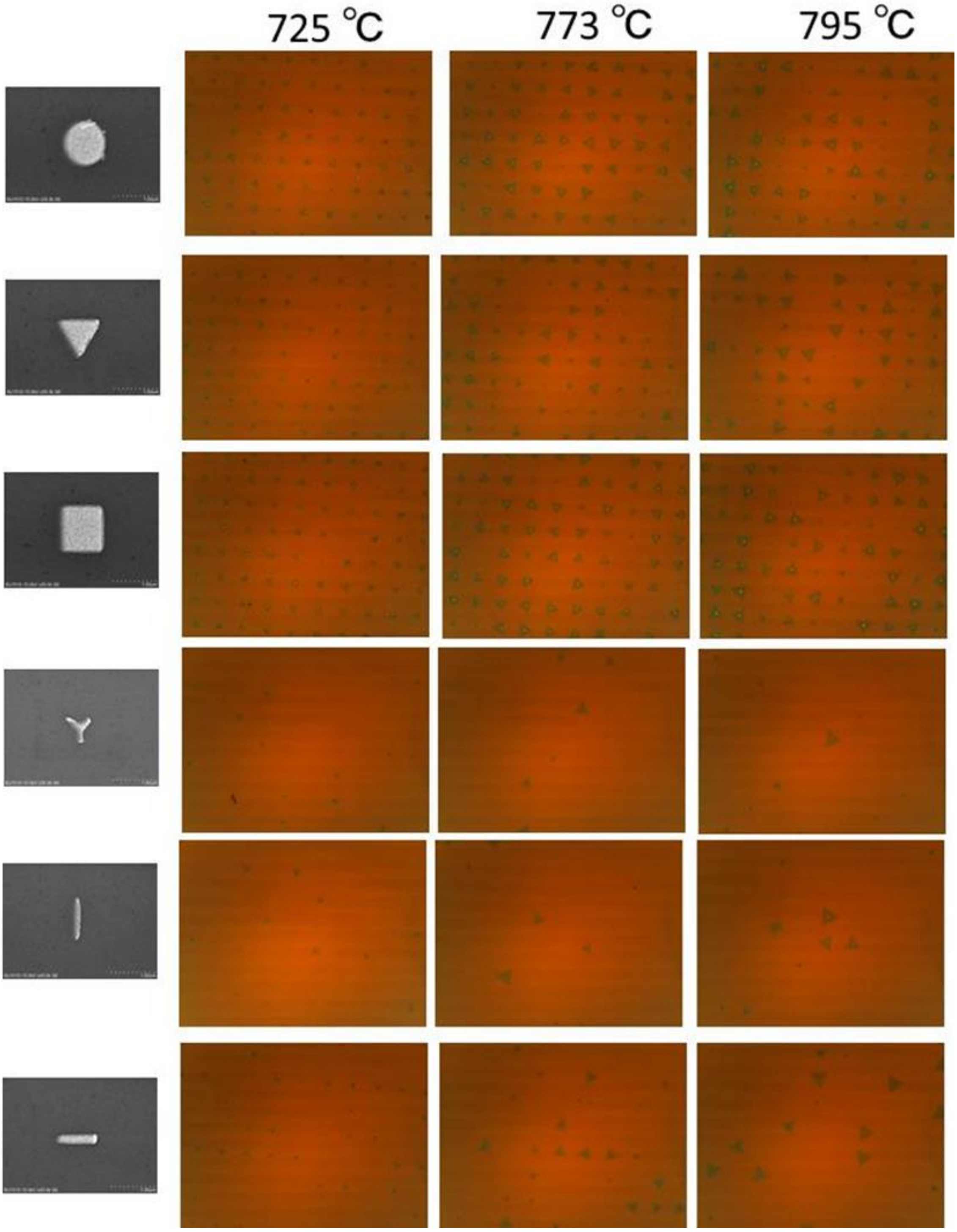

Supplementary Figure 4 │ Effects of Au dot shape. Optical images of WS_2 grown on the Au dot array. Au dot shapes and growth temperatures varied on the substrate in the combinatorial experiment.

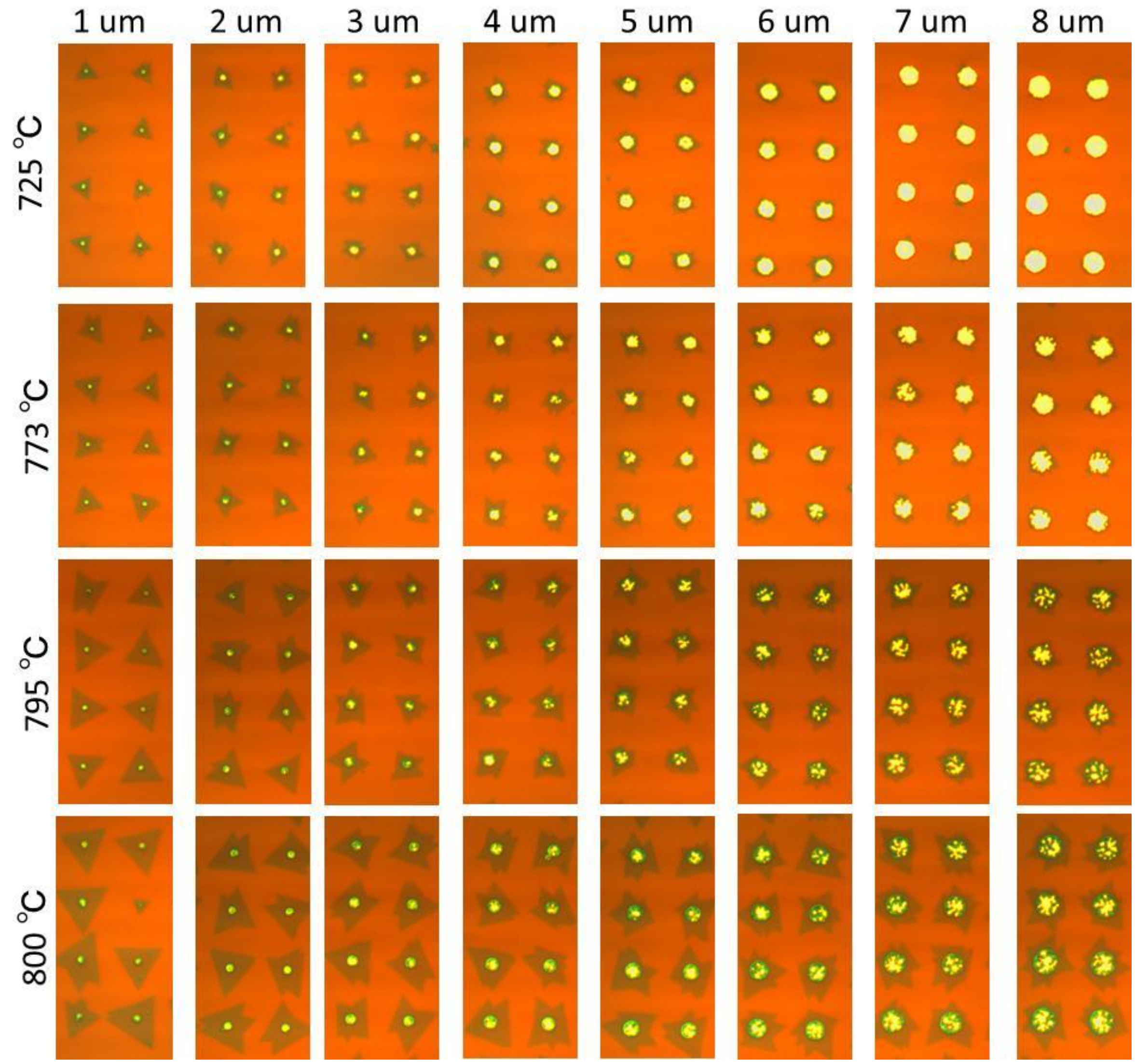

Supplementary Figure 5 | Effects of Au dot size. Optical microscope images of WS_2 grown on the Au dot array. D_{Au} was varied from 1 μm to 8 μm on the substrate. The growth temperature gradient in the CVD system was 725 to 800 °C.

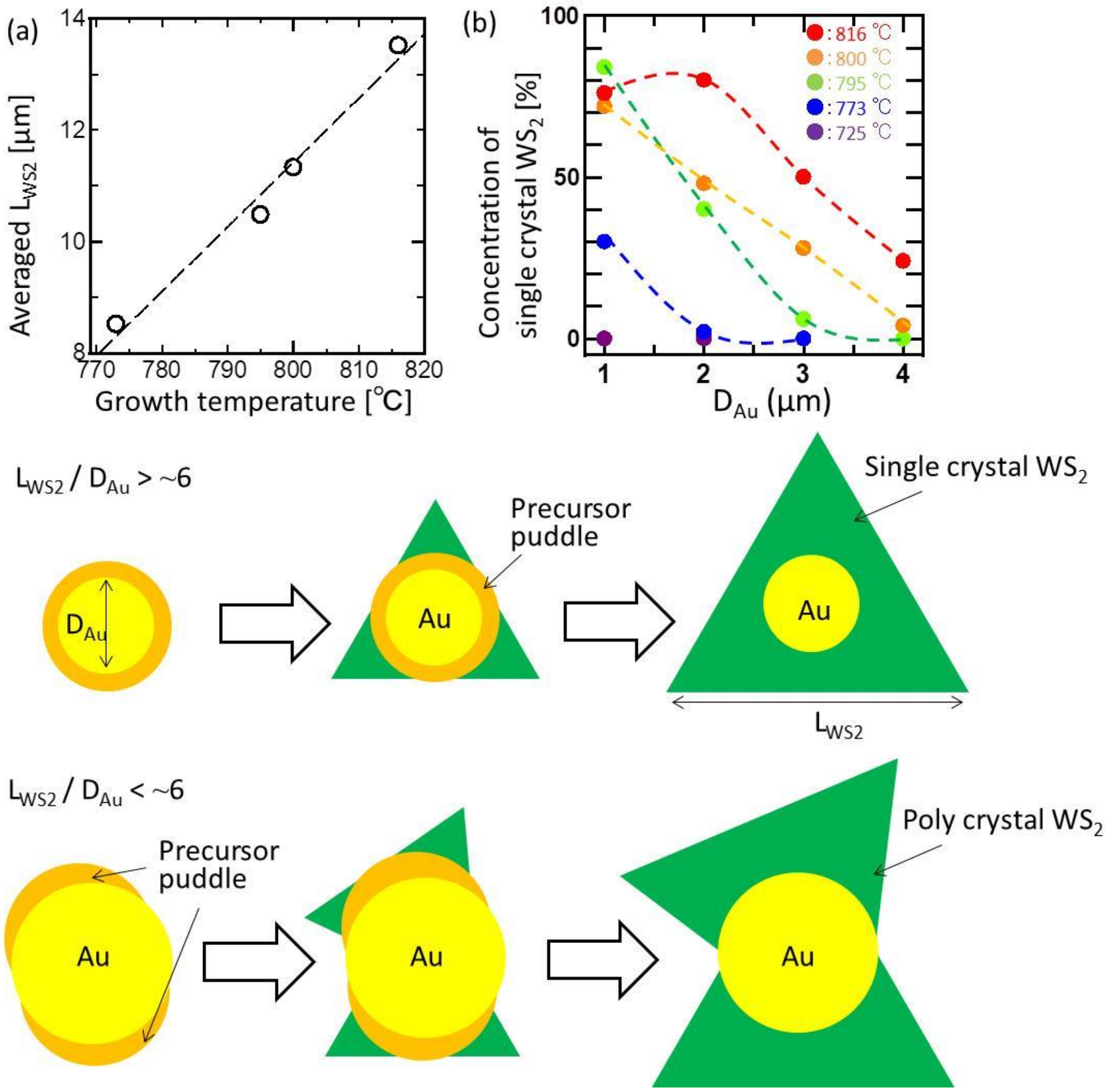

Supplementary Figure 6 │ Effect of D_{Au} on WS_2 single crystal growth. a, Average L_{WS2} as a function of growth temperature. **b**, WS_2 single crystal concentration plotted according to D_{Au} and growth temperature. Schematic model of (**c**) single crystal and (**d**) polycrystalline growth on Au dots.

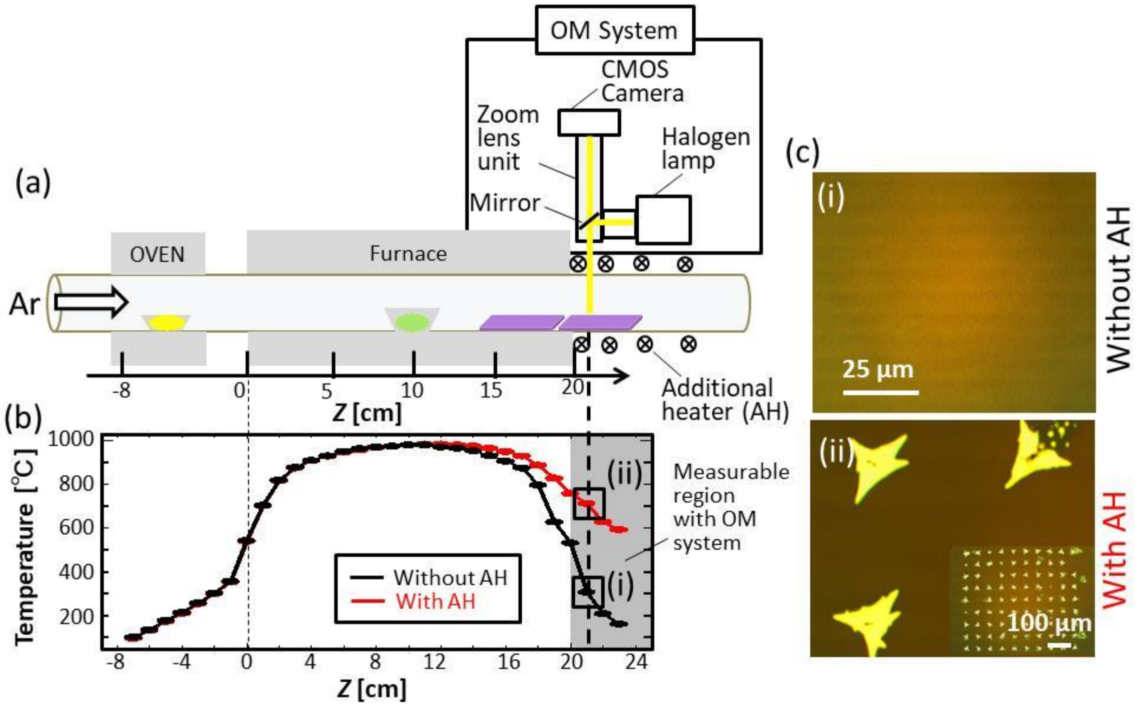

Supplementary Figure 7 │ In-situ monitoring system for WS_2 growth. a, Schematic illustration of in-situ CVD monitoring system. **b**, Temperature profile of the CVD system with (red) and without (black) the additional heater. **c**, Optical microscope images of the substrate (*z* = 22 cm) after CVD (i) without and (ii) with the additional heater.

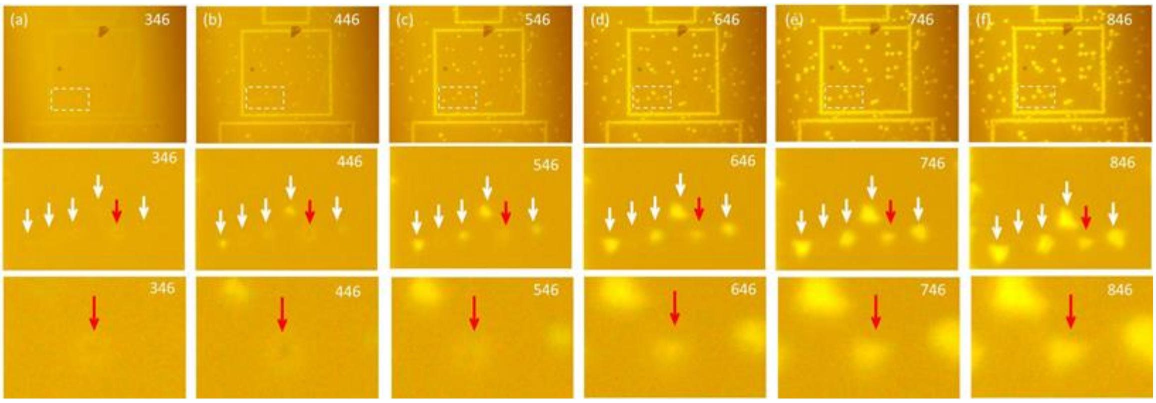

Supplementary Figure 8 │ Detailed results from in-situ monitoring. Low-magnification (upper), mid-level magnification (middle) and high-magnification (bottom) optical microscope images collected during WS_2 growth. Growth temperature: **a**, 346 s, (**b**) 446 s, (**c**) 546 s, (**d**) 646 s, (**e**) 746 s, (**f**) 846 s. The red arrows indicate the specific WS_2 crystals for which droplet nucleation was observed.

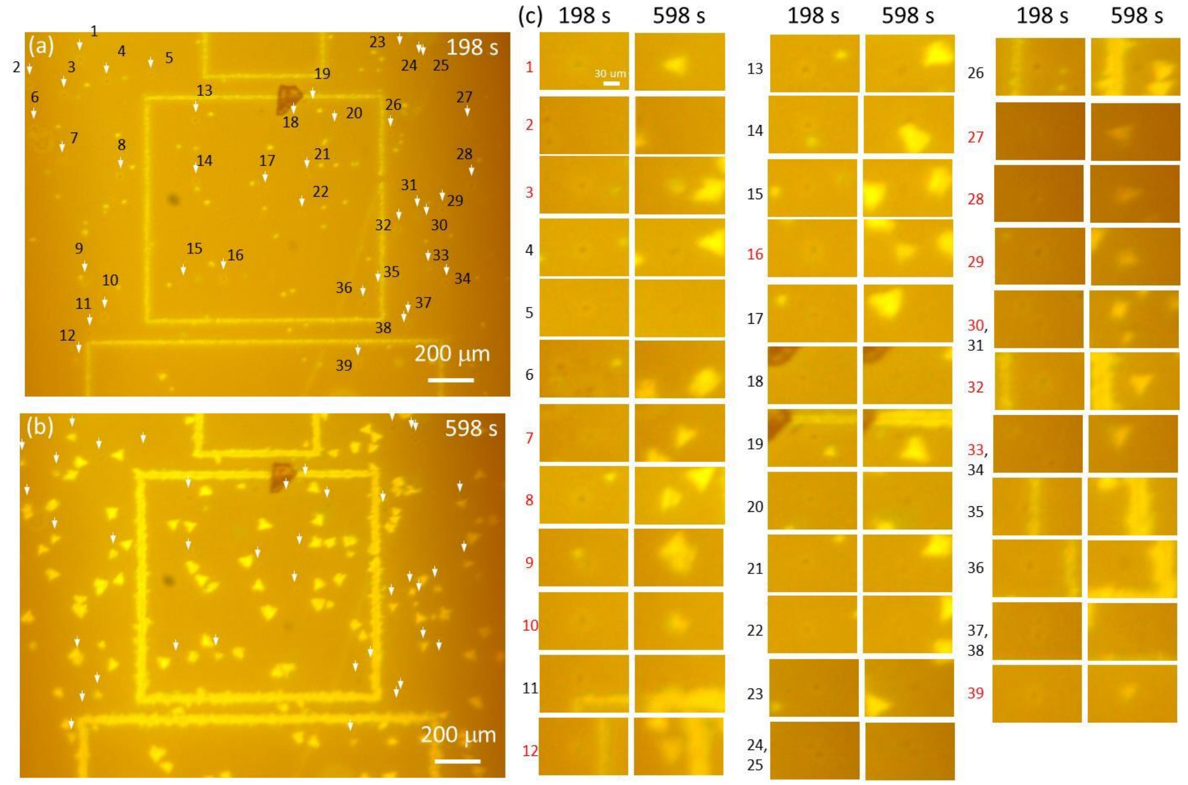

Supplementary Figure 9 │ Transformation from precursor puddle to WS_2. Low-magnification optical microscope images collected during in-situ monitoring of WS_2 growth at (**a**) 198 s and (**b**) 598 s. The small arrows point to (**a**) the precursor puddles and (**b**) the same positions after WS_2 growth. **c**, High-magnification images of precursor puddles observed at 193 s and the final products after 598 s of growth. The numbers correspond to those shown in (**a**). The numbers in red indicate positions where precursor puddles transformed into WS_2 crystals.

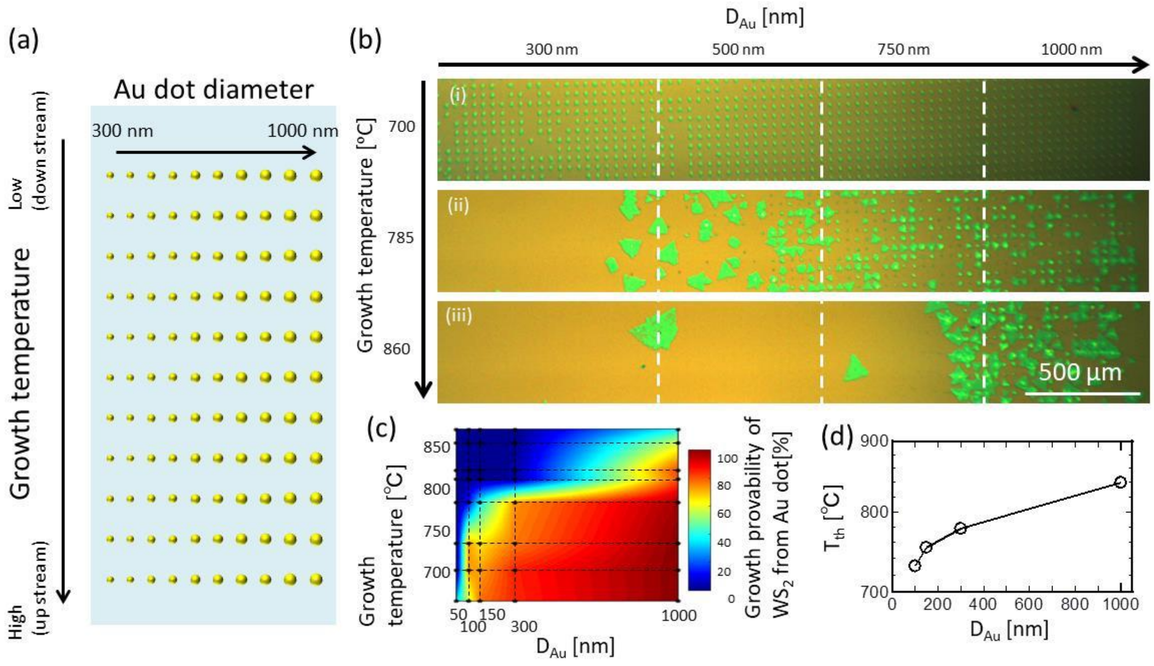

Supplementary Figure 10 │ Combinatorial experiment for position-selective synthesis of WS_2. a, Schematic illustration of the substrate used for the combinatorial experiment. **b**, Optical microscope images of WS_2 grown on the SiO_2 substrate with Au dots at (i) 700 °C, (ii) 785 °C and (iii) 860 °C. D_{Au} of Au dots: 100, 300, 500, 750 and 1000 nm. **c**, Contour plot of WS_2 growth yield on Au dots according to growth temperature and D_{Au}. **d**, Threshold temperature (T_{th}) at which the growth yield of a WS_2 single crystal exceeds 60% as a function of D_{Au}.

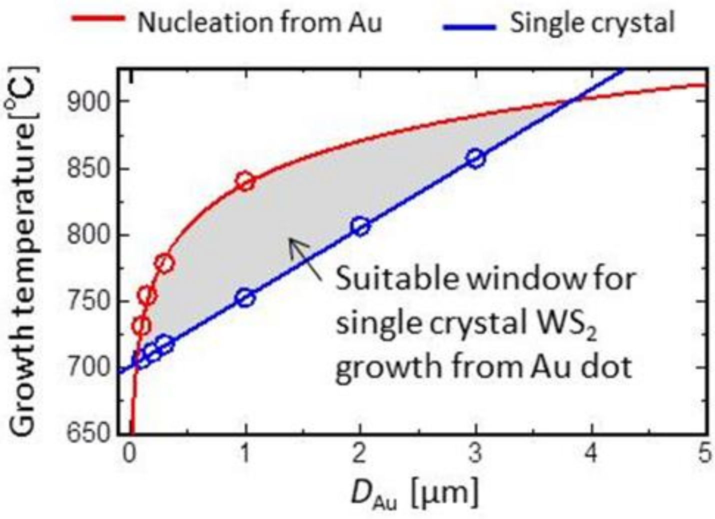

Supplementary Figure 11 │ WS_2 single crystal growth window on Au dots. Correlation between growth temperature and D_{Au} for WS_2 single crystal growth based on experimentally determined nucleation probability (red) and observed single crystal growth (blue).

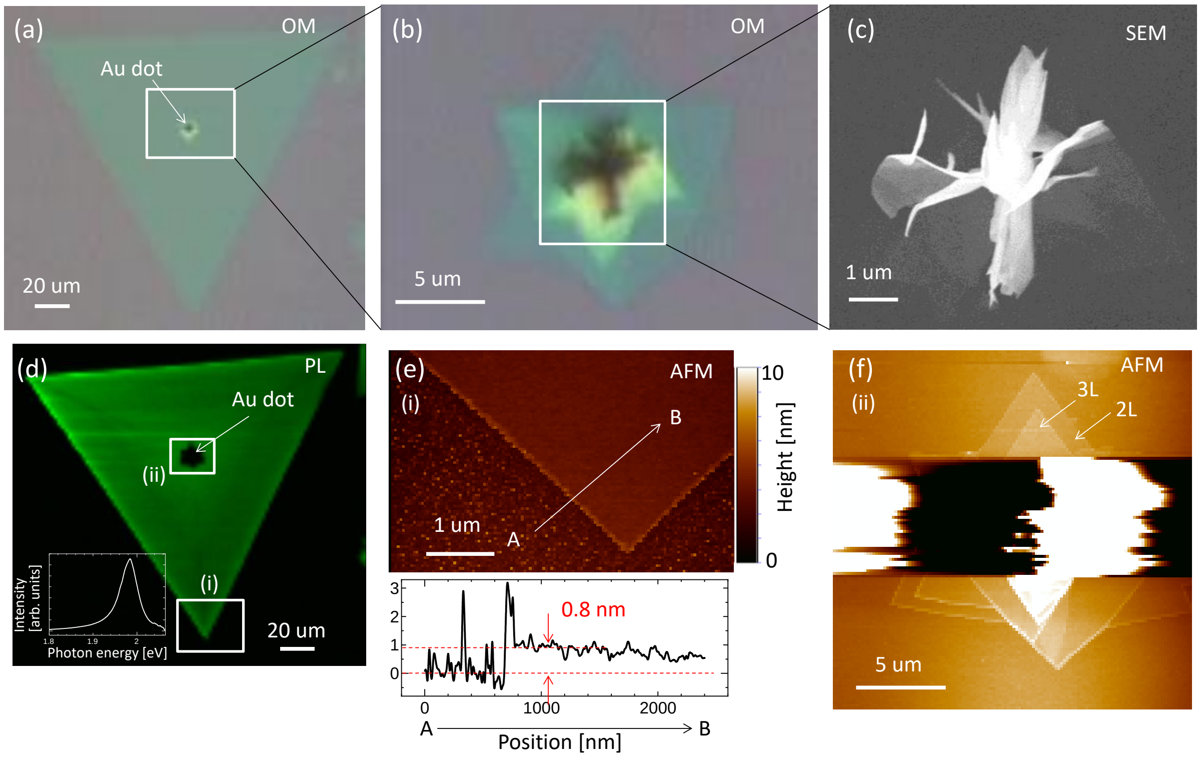

Supplementary Figure 12 │ Anallysis of WS2 near the Au dot region. (a-c) (a,b) Optical microscope and (c) SEM image of (a) whole and (b,c) near the Au dot of monolayer WS2 grown from Au dot. (d) PL intensity mapping of whole WS2 crystal. Inset shows typical PL spectra. (e,f) AFM image of WS2 taken at (e) edge of WS2 (region i in d) and (f) near the Au dot (region ii in d). Bottom of (e) shows line scan of height profile taken from A to B in (e).

Supplementary Table 1| L_d of conventional semiconductor materials.

**Semiconductor Material**	**L_d (μm)**	**T (°C)**	**Ref.**
Ga	0.5	580	[S1]
Ga	2	690	[S2]
Ga	0.5	670	[S3]
Ga	1	560	[S4]
Ga	8	560	[S5]
Ga	10	580	[S6]
Si	0.2	650	[S7]
Si	30	1000	[S8]
SiC	13	1500	[S9]

References

[S1] Morishita, Y. Nomura, Y. Goto, S. and Katayama, Y. Effect of hydrogen on the surface-diffusion length of Ga adatoms during molecularbeam epitaxy. *Appl. Phys. Lett.* **67**, 2500-2502 (1995)

[S2] Nilsson, S. *et al.* Ga adatom migration over a nonplanar substrate during molecular beam epitaxial growth of GaAs/AlGaAs heterostructures. *Appl. Phys. Lett.* **55**, 972–974 (1989).

[S3] Shitara, T. & Nishinaga, T. Surface diffusion length of gallium during MBE growth on the various misoriented GaAs(001) substrates. *Jpn. J. Appl. Phys.* **28**, 1212–1216 (1989).

[S4] Hata, M., Isu, T., Watanabe, A. & Katayama, Y. Real-time observation of molecular beam epitaxy growth on mesa-etched GaAs substrates by scanning microprobe reflection high-energy electron diffraction. *Appl. Phys. Lett.* **56**, 2542–2544 (1990).

[S5] Hata, M., Watanabe, A. & Isu, T. Surface diffusion length observed by in situ scanning microprobe reflection high-energy electron diffraction. *J. Cryst. Growth* **111**, 83–87 (1991).

[S6] Nomura, Y., Morishita, Y., Goto, S., Katayama, Y. & Isu, T. Surface diffusion length of Ga adatoms on $(\bar{1}\bar{1}\bar{1})$B surfaces during molecular beam epitaxy. *Appl. Phys. Lett.* **64**, 1123–1125 (1994).

[S7] Lim, S.-H., Song, S., Park, T., Yoon, E. & Lee, J.-H. Si adatom diffusion on Si (100) surface in selective epitaxial growth of Si. *J. Vac. Sci. Technol. B* **21**, 2388-2392 (2003).

[S8] Nielsen, J.-F., Pelz, J. P., Hibino, H., Hu, C.-W. and Tsong, I. S. T. Enhanced Terrace Stability for Preparation of Step-Free Si(001)-(2x1) Surfaces. *Phys. Rev. Lett.* **87**, 136103-1-4 (2001)

[S9] Kimoto, T. and Matsunami, H. Surface diffusion lengths of adatoms on 6H-SiC{0001} faces in chemical vapor deposition of SiC. *J. Appl. Phys.* **78**, 3132-3137 (1995).
